# Stationary bubble formation and cavity collapse in wedge-shaped hoppers

**DOI:** 10.1038/srep25065

**Published:** 2016-05-03

**Authors:** Yui Yagisawa, Hui Zee Then, Ko Okumura

**Affiliations:** 1 Department of Physics and Soft Matter Center, Ochanomizu University, 2–1–1, Otsuka, Bunkyo-ku, Tokyo 112-8610, Japan

## Abstract

The hourglass is one of the apparatuses familiar to everyone, but reveals intriguing behaviors peculiar to granular materials, and many issues are remained to be explored. In this study, we examined the dynamics of falling sand in a special form of hourglass, i.e., a wedge-shaped hopper, when a suspended granular layer is stabilized to a certain degree. As a result, we found remarkably different dynamic regimes of bubbling and cavity. In the bubbling regime, bubbles of nearly equal size are created in the sand at a regular time interval. In the cavity regime, a cavity grows as sand beads fall before a sudden collapse of the cavity. Bubbling found here is quite visible to a level never discussed in the physics literature and the cavity regime is a novel phase, which is neither continuous, intermittent nor completely blocked phase. We elucidate the physical conditions necessary for the bubbling and cavity regimes and develop simple theories for the regimes to successfully explain the observed phenomena by considering the stability of a suspended granular layer and clogging of granular flow at the outlet of the hopper. The bubbling and cavity regimes could be useful for mixing a fluid with granular materials.

Dynamics of dense granular materials is one of the challenging problems in physics^1^,^2^. For example, recently, dynamic aspects of jamming transitions in granular systems have emerged as an important issue and have received considerable attention both experimentally and theoretically^3^,^4^,^5^,^6^,^7^. Contrary to this, the flow in an hourglass or the discharge of a silo through an orifice is a classic and familiar problem of granular dynamics, but these phenomena still remain an issue actively discussed in recent studies. In general, such granular flow can be continuous, intermittent^8^,^9^, or completely blocked^10^,^11^. In the continuous region, standard views on the hourglass dynamics^12^,^13^,^14^,^15^ have recently been questioned via self-similar dynamics^16^, universal scaling in a pressure transition^17^, and independent control of the velocity and pressure^18^. In the intermittent region, clogging transitions under some agitation (e.g. vibration or random forces) are discussed from a unified viewpoint^19^. In the blocked region, clogging in tilted hoppers is discussed to clarify a phase diagram^20^,^21^ and jamming in a two-dimensional hopper is explained by associating the arch at the orifice with a random walk^22^.

Here, we studied granular flow in an hourglass without circular symmetry (more specifically, wedge-shaped hoppers) when a suspended granular layer could be stabilized to a certain extent, with the aid of cohesion force between sand grains, friction with side walls, and low permeability of the sand layer (which leads to the pressure difference developed in the layer)^8^,^9^. As a result, we found two spontaneous behaviors: stationary formation of bubbles and cavity collapse. We show that these two opposite effects emerge via the interplay between stability of the sand layer and clogging at the orifice. Note that the clogging discussed in the present study is temporal in the sense that the flow recovers without adding external perturbation and is governed by the cohesion between particles unlike in the previous studies^10^,^20^,^21^, and thus quite different from the clogging studied in the previous studies.

The cavity regime cannot be categorized into any previously known phases: it is neither continuous, intermittent nor completely blocked phases. Any systematic physical study on this original regime using a small-scale laboratory setup have never been discussed in the physics literature. We show that this intriguing phenomenon can be understood by the balance between the gravitational and frictional forces.

The bubbling reported here is quite visible to a level never reported in the literature. Although a similar phenomenon is reported^8^,^9^ (a density wave or flow pattern is also reported for much larger grains in hoppers by use of X-Ray radiography^23^), the bubbles are much less visible. In fact, what are called “bubbles” in the previous study^9^ are quite localized near the orifice: the “bubble” sizes are typically about 6 mm and they “rise” at most 2–3 mm in an hourglass with the orifice of size 3.7 mm. In contrast, in the present case, although the orifice size is smaller and is typically 2 mm, the bubble sizes can be more than 10 mm and they can rise more than 70 mm (they typically rise to the top surface of the sand layer). In addition, in the present case, more than one bubble can coexist at the same time, as often seen when bubbles are created not in sand but in liquid.

Since the bubbling in the present study is so clear as if it appeared in liquid, it is possible to study the bubble size and the rising speed in detail. As a result, we elucidate the dependence of the period of bubble nucleation on the geometry of hourglass and clarify the importance of clogging in the periodic motion.

Note that, although visible bubbling in a granular layer has been discussed for fluidized beds^24^,^25^, bubbles in the context emerge as a result of an externally imposed fluid flow. In contrast, bubbles discussed here appear spontaneously and repeatedly in a hourglass without any externally imposed flow.

## Results

**Figure 1:**
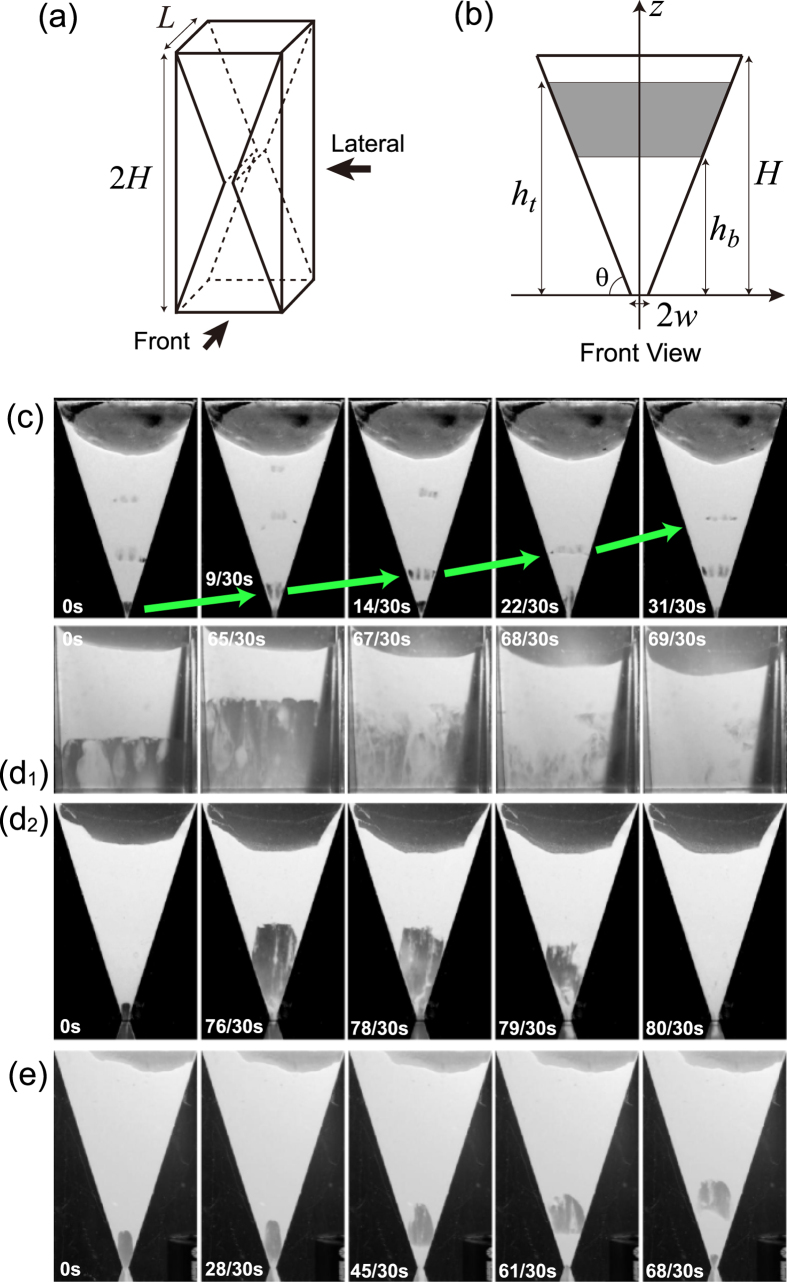
(**a**) Illustration of an hourglass without circular symmetry. The front and lateral sides are defined as indicated by the arrows. (**b**) The front view of the upper half of the acrylic cell in (**a**). The position of the top and bottom surfaces of the sand layer measured from the outlet is called *h*_*t*_ and *h*_*b*_, respectively. The slope is called *α* (=tan *θ*). (**c**) Series of snapshots taken from the front side of the cell in the bubbling regime where the diameter of glass beads *d*, the cell thickness *L*, the half size of the outlet *w* and the angle *θ* are given as 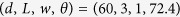 in the units *μ*m, mm, mm, and deg., respectively. (**d**_**1**_) Snapshots taken from the lateral side in the cavity regime for 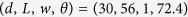 in the same units. (**d**_**2**_) Snapshots taken from the front side in the cavity regime for 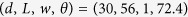. (**e**) Snapshots from the front side in the intermediate regime for 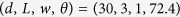. Corresponding movies are available for (**c**,**d**_**1**_), as Supporting Information video files.

We use a transparent closed cell of height 2*H*, thickness *L*, and slope *α* (that defines the angle *θ* via *α* = tan *θ*), as shown in Fig. 1(a,b). The cell is half-filled with glass beads, which are nearly monodisperse. The outlet width 2*w* of the cell is much larger than the average diameter *d* of the beads. By rotating the cell upside down, all the glass beads in the cell that are initially located above the outlet start falling down through the outlet due to gravity.

### Dynamic regimes

Depending on the parameter set, *d*, *L*, *θ*, and *w*, the dynamics are categorized into three regimes. (1) Bubbling regime: small air bubbles are regularly created at the outlet and goes up to disappear, as shown in Fig. 1(c). (2) Cavity regime: a large cavity starts to grow from the outlet to finally collapse, as seen in Fig. 1(d1,d_2_). (3) Intermediate regime: a cavity grows upwards first but later glass beads accumulate near the clogged outlet, leading to a “rising cavity,” as in Fig. 1(e).

**Figure 2:**
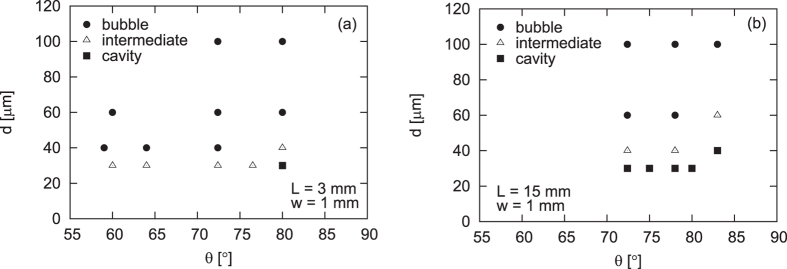
Phase diagram as a function of *θ* and *d* obtained from thin (**a**) and thick (**b**) cells.

The phase diagrams in Fig. 2 can be physically understood by considering the following points:

1. The dynamic regime is determined as a result of competition of the stability of the bottom free surface of the sand layer and the clogging at the orifice. A cavity can keep growing only when the free surface is stable and only when clogging is difficult to occur. Bubbling occurs under the opposite conditions (low stability and easy clogging): a bubble looks as if to “rise up” in the granular medium, as a result of the collapse of the top part (due to low stability) followed by the accumulation of sand (due to clogging).
2. The stability is increased when particles are fine. This is because the cohesive force between particles becomes large when the sand is fine. This is well-known at least empirically and explained frequently in terms of capillary bridges between sand particles originating from a tiny amount of water located at the contacts between grains^1^,^2^. The ratio of the capillary force *kγd* to the gravitational force *ρgd*^3^ for a particle of diameter *d*, which scales as *k*(*l*/*d*)^2^, becomes larger when particle becomes small. Here, *γ*, *ρ* and *g* are the surface tension of water, the density of the particle, and the gravitational acceleration, defining the capillary length 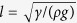. In the atmospheric humidity, the numerical coefficient *k*, which reflects the amount of water at the contacts of particles, would be very small. However, for example, when *d* is 30 *μ*m, the ratio *k*(*l*/*d*)^2^ could become comparable to unity because *l* is about 3 mm: for small particles, the cohesive force can excel the gravitational force. Another reason for the high stability of fine particles is the small permeability of the sand layer formed by fine grains. This will help create pressure difference in the sand layer, contributing the stability. This point will be discussed in more detail in the next two sections.
3. The clogging occurs easily when the slope is small and when the cell is thin. Easy clogging for small slopes is reasonable: the smaller the slope is, the more concentrated towards the outlet the falling sand beads are. Easy clogging for thin cells (small *L*) is understandable from snapshots in Fig. 1(d1), as explained as follows. In each snapshot, we can observe streams of falling sand whose width and numbers are changing with time (for example, in the left-most snapshot in Fig. 1(d1) we recognize 4 streams three of which have almost the same width while the left-most stream is the widest). When the cell thickness is small and comparable to a typical width of such streams, the flow flux averaged by the orifice area is essentially the same with the flow flux of the stream. However, when the cell thickness is large and several streams are observed with some spacing between them as in Fig. 1(d1), the average flux at the orifice is smaller than the flow flux of the streams. As a result, we expect that clogging is more difficult to occur in a cell of large thickness because of the smaller average flux (Note that the stability is not completely independent from the clogging: The flux is correlated with the stability of the free surface so that high (low) stability implies that clogging is difficult (easy)). In summary, clogging is easy to occur for thin cells whose slope is small.

On the basis of the above points (1)–(3), the phase diagrams in Fig. 2 can be physically understood: The cavity regime should be observed only when the diameter *d* of sand is small (for strong cohesion and low permeability), the cell thickness *L* is large, and the angle *θ* is large (for clogging to be difficult), whereas the bubbling regime tends to be seen under the opposite conditions, i.e., when *d* is large, *L* is small, and *θ* is small. These tendencies are clearly confirmed in Fig. 2 for *w* = 1 mm.

### Bubbling regime

In the bubbling regime, the *n*th bubble is created as follows. First, a cavity is nucleated at the outlet at time 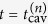 and, then the cavity grows as beads keep falling (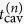 is the nucleation time of the *n*th cavity). At time 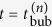, clogging occurs at the outlet, closing the outlet: this is the moment of creation of a bubble (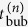 is the creation time of the *n*th bubble). The *n*th bubble thus created rises up in the granular medium upwards, whereas after a short waiting time 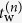 (i.e., at time 
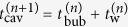
) the next (*n* + 1)th cavity is nucleated at the clogged part at the bottom, which leads to the next bubble.

**Figure 3:**
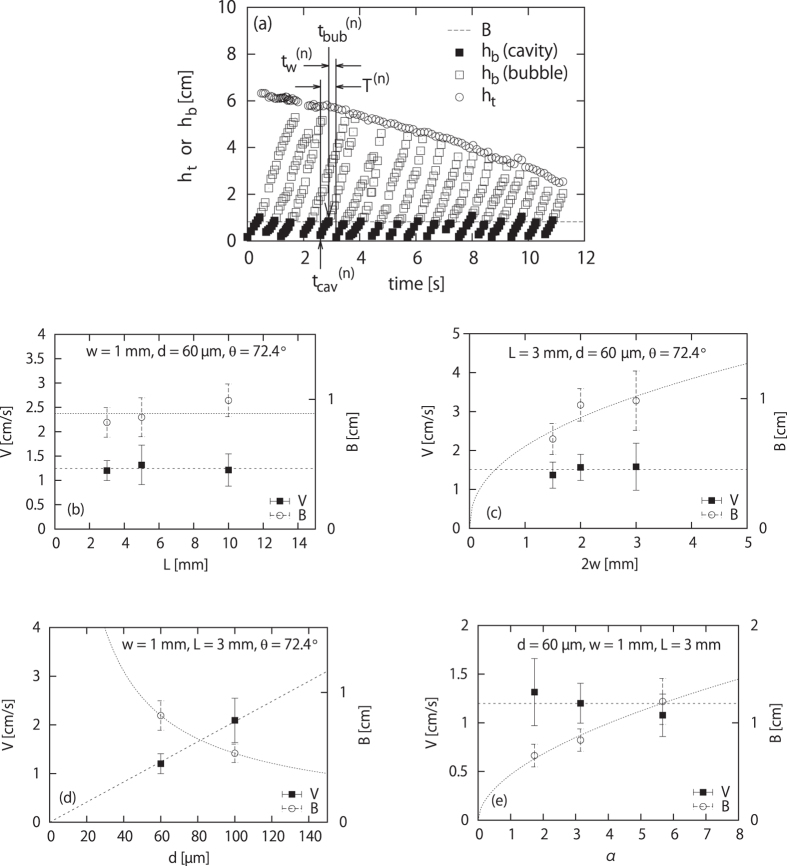
(**a**) *h*_*t*_ and *h*_*b*_ (see Fig. 1(b)) vs time in the bubble regime for *d* = 60 *μ*m, *L* = 3 mm, *w* = 1 mm, and *θ* = 72.4°. The quantities 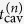, 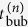, and 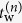 are the nucleation time of the *n*th cavity, the creation time of the *n*th bubble, and the waiting time for the (*n* + 1) th cavity, respectively. The horizontal dashed line shows the average value *B* of the bubble size *B*^(*n*)^ at the time of bubble creation. (**b**) *V* and *B* vs *L* for a given *w*, *d*, and *θ*. (**c**) *V* and *B* vs 2*w* for a given *L*, *d*, and *θ*. (**d**) *V* and *B* vs *d* for a given *w*, *L*, and *θ*. (**d**) *V* and *B* vs *α* (=tan *θ*) for a given *L*, *d*, and *w*. The fitting curves for (**c**–**e**) are given as, 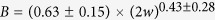, *B* = 27*d*^−0.85^ and *V* = (0.021 ± 0.000) × *d*, and 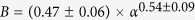, respectively.

To quantify this dynamics, the positions of the top and bottom surfaces of the sand layer, *h*_*t*_ and *h*_*b*_ (defined in Fig. 1(b)), are plotted in Fig. 3(a) for a parameter set in the bubbling regime. The position of the bottom is marked by squares: Filled and open squares correspond to the data before and after the clogging at 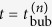, respectively. In other words, filled and open squares stand for the top surface of a cavity and that of a bubble, respectively.

Figure 3(a) shows a repeated or regular dynamics: the time interval between consecutive bubble creations, the rising speed of bubbles, and the initial bubble size are almost constant. As seen in Fig. 3(a), the cavity nucleation (at time 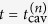) and the bubble formation (at time 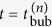) occur regularly and the interval defined as 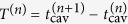 is almost a constant *T*. The growing speed of the *n*th cavity 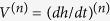 defined for the interval from 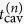 to 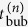 is also almost a constant *V*, whereas the velocity *V*^(*n*)^ is comparable to the rising speed of the *n*th bubble defined after 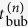. This means that the initial bubble size *B*^(*n*)^, defined as 
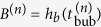
, is also a constant *B*, as seen in Fig. 3(a), whereas the following equation holds:


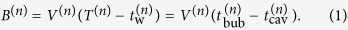


The *n* - independence of the growing speed *V*^(*n*)^ = (*dh*_*b*_/*dt*)^(*n*)^ is interpreted as follows. This quantity essentially corresponds to the collapsing speed of the bottom surface (at height *z* = *h*_*b*_) of the suspended granular layer, which can be determined as follows. By introducing a life time *τ* of a sand particle after the moment the sand particle is exposed to the sand-air interface (positioned at height *z* = *h*_*b*_), we simply obtain *the growing speed of a cavity or the rising speed of a bubble*,


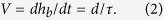


As seen in Fig. 3(a), the slope (*dh*_*b*_/*dt*)^(*n*)^ is almost independent of *n* and *h*_*b*_ (although the slope has a slight tendency to increase with *h*_*b*_). This means that the life time *τ* and the rising speed of bubbles are also independent of *n* and *h*_*b*_ (although *τ* has a slight tendency to decrease with *h*_*b*_ and the average rising speed of bubbles has a slight tendency to increase with *h*_*b*_). The physical nature of *τ* will be explored in Discussion.

The *n* - independence of the initial bubble size *B*^(*n*)^ is interpreted as follows. We introduce the two dimensional volume fraction of sand beads in the layer *ϕ*, which is practically assumed to be the random closed packing value *ϕ*_*c*_ as an approximation. The volume flux created at the bottom surface of the sand layer, *Q*_*b*_, is given as *Q*_*b*_ = *ϕSV*, where *S* is the surface area of the free surface at *z* = *h*_*b*_ (note that in the bubbling regime, *L* is smaller than the typical width of streams of flowing sand mentioned above) and thus given by *S* = *Lh*_*b*_/*α* (when *w* is small). The maximum flux at the outlet *Q*_0_ is estimated as *Q*_0_ = *ϕ*_*c*_*S*_0_*V*_0_, with the section area *S*_0_ = 2*wL* of the cell at the outlet (*z* = 0) (see Fig. 1(b)) and the velocity at the outlet *V*_0_. This velocity is determined by 
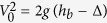
, where *g*Δ expresses a small energy dissipation (per unit mass) for sand beads falling off from the surface after gliding on the cell wall of the slope *α*. By noting that 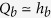 and 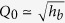 (Δ is generally small in the experiments), we see that, just after the nucleation of a cavity, the clogging does not occur, because *Q*_*b*_ < *Q*_0_ (i.e., 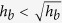) for small *h*_*b*_. Then, the critical condition for clogging is given by *Q*_*b*_ = *Q*_*o*_. The value of *h*_*b*_ obtained by solving this critical condition in favor of *h*_*b*_ corresponds to the bubble size *B* (This is because the size of *h*_*b*_ at the creation of the bubble is the definition of the initial size of the bubble, *B*, as defined above). From this clogging condition and Eq. (2), we obtain


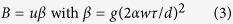


where 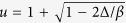 with 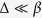. This explains why the horizontal dashed line in Fig. 3(a) gives a well-defined average value *B*, that is, why 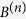 is almost independent of *n*. Since the waiting time 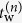, which corresponds to the reorganization time for an once packed granular layer formed near the outlet to destabilize (see below for more details), is expected to be *n*-independent, the *n*-independence of *V* and *B* justify the n-independence of *T*^(*n*)^ [see Eq. (1)].

The agreement of the above theory with experiment is seen in different ways in Fig. 3(b–e), which supports Eqs (2) and (3). Note that *B* is predicted as an increasing function of *αw*/*d* from Eq. (3) as understood from the approximate expression *B* ≃ 2*β* obtained in the limit Δ ≪ *β*. Then all the following behaviors shown in Fig. 3(b–e) are consistent with Eqs (2) and (3), if the dependence of *τ* on *d* is relatively weak. (1) Figure 3(b) shows that *V* and *B* are independent of *L*. (2) Figure 3(c) shows that *V* is independent of *w* and that *B* increases with *w*. (3) Figure 3(d) shows that *V* increases with *d* and that *B* decreases with *d*. (4) Figure 3(e) shows that *V* is independent of *α* and that *B* increases with *α*.

**Figure 4:**
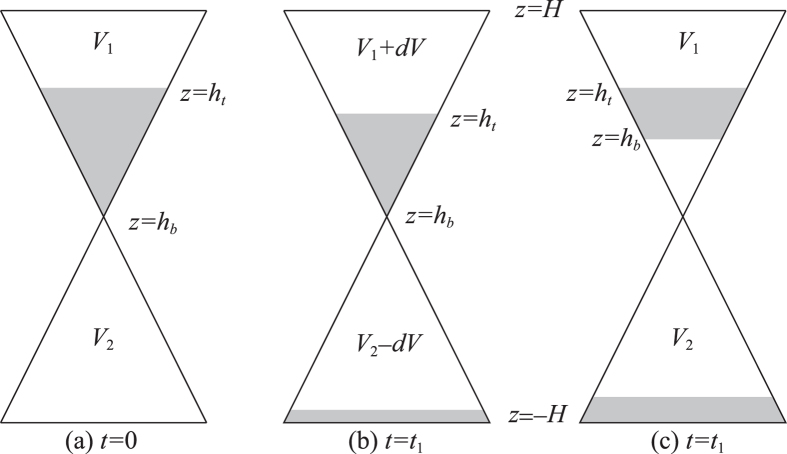
Creation and non-creation of air pressure difference in the chambers of an hourglass. The shaded areas stand for the regions occupied by the sand, which is here considered as completely impermeable. In the change from (**a**) at the time *t* = 0 to (**b**) at the time *t* = *t*_1_, the pressure difference is created because of the changes in volume of the air in the chambers (from the volumes *V*_1_ and *V*_2_ to *V*_1_ + *dV* and *V*_2_ − *dV*). In the changes from the state in (**a**) to the state in (**c**), the pressure difference is not created, corresponding (nearly) to the case in the cavity regime.

The importance of a weak and localized pressure difference created near the orifice was clearly shown in the intermittent regime^8^,^9^. They showed the existence of the active phase in which the flow through the orifice is maintained and the pressure difference increases with the increase in *h*_*b*_ till the bubble disappears. This active phase is followed by the inactive phase in which the flow through the orifice is stopped and the pressure difference decreases till the creation of the next cavity. During the active phase, because of the transfer of sand from the upper to lower chamber, the pressure in the sand layer near the orifice becomes larger than the pressure in the upper chamber due to the compression and expansion of air in the lower and upper chambers, respectively, provided that the chambers are closed (Note however this is true only when *h*_*t*_ decreases as *h*_*b*_ increases; see Fig. 4). This pressure difference helps stabilize the bottom of the sand layer, leading to a complete stop of the flow, i.e., to an initiation of the inactive phase. During the inactive phase the pressure differences thus created is gradually reduced by the permeation of air in the sand layer till the bottom of the sand destabilizes again, leading to an intermittent dynamics. They developed a theory describing the duration of inactive phase *T*_*i*_ on the basis of the Darcy law on the permeation of viscous fluid in porous materials and confirmed the theory by experiments. However, they did not give a theory describing the duration of the active phase *T*_*a*_ although they indicated the importance of clogging, by explicitly saying^9^ “the plug was created when the flux of particles coming from the free-fall arch (corresponding to the bottom surface of the sand layer at *z* = *h*_*b*_) was too large to pass through the orifice rapidly.” Note here the following points for this series of study^8^,^9^: (1) They claimed that the period *T*_*i*_ + *T*_*a*_ was quite robust and independent of the grain size and explained the characteristic order of the period by the Darcy dynamics in the first series of study^8^; (2) However, in the second series of study^9^ the view on the robustness was modified and they suggested the importance of clogging in the active phase as above but without developing a theory.

Bubbling in the present study and in the previous studies^8^,^9^ are quite different. The size of bubbles is much larger and the life time of bubbles is much longer in the present study. A marked difference is that bubble in the present cases never disappears before they reach the top of the sand layer *h*_*t*_. In addition, more than one bubble can coexist in the sand layer in the present case.

However, we consider that the pressure difference is still important in the present bubbling regime, which is an intermittent regime as in the previous studies^8^,^9^, although detailed effects should be rather different (see below). For example, the period *T*_*i*_ in the previous study, in which the pressure difference is reduced, corresponds to the waiting time *t*_w_ in the present study. Our theoretical arguments based on the clogging condition given above provides an estimate for the counterpart of *T*_*a*_ in which period the pressure difference is increased, whereas any explicit estimates are not available in the previous studies^8^,^9^. The counterpart of *T*_*a*_ is here defined as 
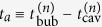
, through the relation 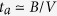. This expression implies that the time *t*_*a*_ increases with *α* and *w* and decreases with *d* (see Equations (2) and (3)). We have confirmed that this is indeed the case in our experiments on the basis of the data shown in Fig. 3(b–e).

Note that the dependence of *t*_*a*_ on *d* in the present study is the opposite to that of *T*_*a*_ on *d*: *t*_*a*_ decreases with *d* whereas *T*_*a*_ increases with *d*. This is a clear experimental fact while the dependence of *t*_*a*_ on *d* is explained by the present theory. This suggests that although the physical mechanisms of bubbling in the present and previous studies are conceptually similar but the details are quite different.

### Cavity regime

**Figure 5:**
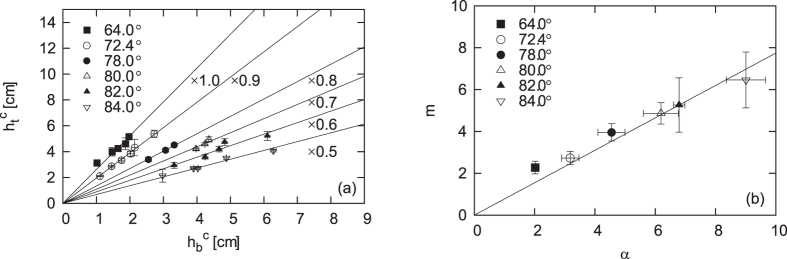
(**a**) 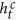 vs 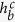, i.e., *h*_*t*_ vs *h*_*b*_ (See Fig. 1(b)) at the moment of the collapse of a cavity in the cavity regime, for various *θ*, i.e., *α* = tan *θ* with *L* = 56 mm, 2*w* = 2 mm and *d* = 30 *μ*m. The data for a fixed *θ* (or *α*) is on a straight line as predicted. To avoid overlap, the values of 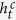 in each series are scaled as suggested on the plot. (**b**) *m* vs *α*. The quantity *m* is here obtained experimentally from the slope of the line fitting the 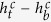 plot (without the rescaling for avoiding the overlap). The data are on the straight line as predicted, with the slope predicts the friction coefficient *μ* between sand particles and cell walls.

In Fig. 5(a), the sudden collapse of the cavity is quantified by plotting *h*_*t*_ as a function of *h*_*b*_ at the moment of collapse; the critical values are respectively denoted 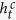 and 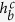 in the plot.

This linear relation 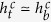 for various conditions shown in Fig. 5(a) can be explained by the condition that the cavity collapse occurs when gravitational force *F*_*g*_ acting on the sand layer exceeds friction force *F*_fric_ acting on the layer from the cell walls. The force *F*_*g*_ is simply estimated by 
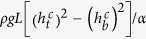
 with *ρ* representing the density of the granular layer. The force *F*_fric_ is estimated as 
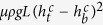
 when *L* ≫ *H* as shown in Methods. When a cavity starts growing, the condition 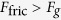 holds. As the cavity becomes larger, both forces decrease but the gravitational force decreases more slowly. As a result, the condition 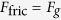 is satisfied in the end, resulting in the collapse of the cavity. This last condition for *L* ≫ *H* gives


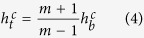


with


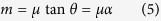


This equation explains the linear relation shown in Fig. 5(a) (All the data in Fig. 5 are obtained for *L* = 56 mm, which satisfies the necessary condition *L* ≫ *H* for Equation (4) to be valid). From the slope of the linear fitting line that should be equal to 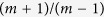, we obtain *m* as a function of *α*, as shown in Fig. 5(b). As predicted in Eq. (5), *m* is linearly dependent on *α*, from which the friction coefficient is obtained as 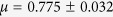. This value also justifies our theory because this value is a reasonable value as a friction coefficient.

The effect of pressure gradient discussed for an intermittent regime in the previous studies^8^,^9^ may play an essential role also in the cavity regime for the stabilization of the suspended sand layer although the cavity regime is not an intermittent regime. However, the collapse condition given in Eq. (4) is still predominantly determined not by the pressure difference but by the friction in the present cavity regime. There are several reasons for this as follows:

1. The pressure difference that would be accumulated if the sand layer were completely impermeable seems to be rather small. This is because, during the cavity formation in which the height *h*_*b*_ is increasing, the height *h*_*t*_ is almost fixed to the initial position, as illustrated in Fig. 4(c). Note that if *h*_*t*_ is completely fixed and the sand layer is completely impermeable, the pressure difference should not be created at all. This is because, in such a case, as illustrated in Fig. 4, there is no change in volume for the air in the space above the sand layer (i.e., in the region, *H* > *z* > *h*_*t*_), and no volume change occurs also for the air in the space below the sand layer and above the sand accumulated at the bottom of the lower chamber (i. e., in the region, 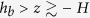): air is neither compressed nor expanded.
2. Even if a small pressure difference is created as suggested in (1), the pressure difference seems to be considerably reduced in reality during the formation of the cavity. This is experimentally supported in two ways. (a) Careful observation near the top surface of the sand layer (at the position 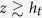) reveals that sand particles are vigorously ejected upwards because of air flow coming out from the sand layer, which obviously relaxes the pressure gradient. (b) As soon as *h*_*b*_ approaches the critical value, the cavity collapses without any observable delay; If the sand layer were predominantly supported by the pressure differences accumulated before reaching the critical state, the collapse would occur only after a delay time in which the pressure gradient would be reduced to some extent by the permeation of air through the sand layer; In fact, in the previous work^9^ in which the effect of pressure gradient is important, they observed such a waiting time *T*_*i*_ of the order of a few seconds. The effect of air permeation during the cavity formation, described in (a), obviously helps stabilize the sand layer because of the viscous drag due to air, especially because the permeability becomes smaller when the grain size is small. However, the fact that the pressure gradient seems almost completely relaxed by the time of the collapse (if otherwise, the delay time should be noticeable because of the small permeability), as discussed in (b), implies that the collapse condition cannot be strongly affected by the pressure gradient.
3. The collapse condition governed by the gravitational and friction force can be dimensionally justified. The two forces acting on the suspended granular layer are given both as *ρgLH*^2^ as implied above because *h*_*t*_ and *h*_*b*_ are roughly of the same order of magnitude as *H*. These estimates elucidate why these two forces can balance with each other to give the collapse condition. On the contrary, the force on the layer that originates from the pressure difference between two chambers scales as 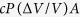, where Δ*V* is very small as implied in (1) and the numerical coefficient *c* is also very small as suggested in (2). Here, *P* is the initial pressure of the chambers and *A* is a characteristic area of the layer, whereas *V* and Δ*V* are a characteristic volume of chambers and changes in them, respectively. Since Δ*V*/*V* and *A* roughly scale as *δ*/*H* and *LH*, respectively, the ratio of the pressure force to the gravitational or frictional force scales as 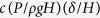. Here, *δ* is the downwards shift of *h*_*t*_ during the cavity formation, which produces the pressure gradient. As mentioned in (1), *δ* is significantly small in the cavity regime. Thus, the ratio 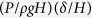 is typically of the order of unity. This justifies why the pressure effect is less dominant because *c* is very small due to the effect of relaxation described in (2).
4. The two independent checks for the agreement between theory and experiment shown in Fig. 5(a,b) strongly support that the collapse of the cavity is properly described by the balance between the gravitational and frictional forces. If the collapse condition is governed not by the friction force but by the pressure difference, we expect that neither Eq. (4) nor Eq. (5) are correct. In reality, both Eq. (4) and Eq. (5) are well confirmed as shown in Fig. 5(a,b), respectively.

## Discussion

At this point, we discuss crude estimates for *τ*, although a better physical understanding of the life time *τ* possibly related to a reorganization time of granular structure will be an important issue to be resolved in the future. One possibility is based on the free fall motion of a grain: this gives 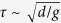. Another possibility is based on viscous drag by the capillary bridges at the contacts: writing the balance between the viscous and gravitational force as 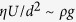, with the viscosity of water being *η* and with the characteristic velocity *U* scaling as *d*/*τ*, we obtain 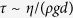. Both crude estimates give similar orders of magnitude: *τ* ~ ms. This is comparable to *τ* estimated by *V* and *d* on the basis of Eq. (2). In addition, both estimates with Eqs. (2) and (3) are consistent with qualitative behaviors of *V* and *B* in Fig. 3 (d).

While humidity affects *τ* as indicated above, the precise control of humidity is experimentally difficult. For example, the data in Fig. 3(a) can be obtained within approximately 10 seconds, so that we observe a well defined *V*, *B*, and *T* in Fig. 3(a). However, the data in Fig. 3(b–d) cannot be obtained in such a short duration and in fact obtained within several hours in the same room. Because of this non-precise control of humidity, we should be satisfied not by a quantitative but by qualitative agreement between theory and experiment in Fig. 3(b–d).

However, we expect the humidity dependence is not too strong. This is because of the following reasons: (1) The bubbling regime appears for larger grains so that the effect of cohesion should be relatively weak in the bubbling regime; (2) The qualitative agreement between theory and experiment in the bubbling regime was obtained, in spite of the non-precise control of humidity.

The reason we observed bubbling and cavity collapse in a hopper that are quite visible to a level never reported in the literature may be the following. The key factor that we successfully observed spontaneous bubbling and cavity collapse in a hopper is the stabilization of a suspended granular layer. When the bubbling regime is observed, the cell thickness is relatively small and the cell is quasi-two dimensional. This helps stabilize a suspended layer because the layer is sandwiched in a small gap between the cell walls, to create a cavity at the outlet, which is soon closed by clogging. When the cavity collapse is observed, the cell thickness is relatively large. In this case, the cohesion force between small sand particles helps stabilize a suspended layer, to create a cavity at the outlet, which is not soon closed because clogging is harder to occur when the cell thickness is large. These stabilization conditions have not been well satisfied in most of the previous physical studies.

However, a phenomenon similar to the one in our bubbling regime are reported^8^,^9^ as already mentioned, in which the importance of the pressure gradient is highlighted. In this respect, the present study provides the following perspective. The pressure gradient is especially important in intermittent regimes, which includes the bubbling regime of the present study. In intermittent regimes, we can recognize two phases: the active phase in which the flow through the orifice is maintained and the inactive phase in which the flow is stopped. At least conceptually (although the details are dependent on experimental parameters such as the geometry of hoppers and the size of grains), the characteristic time scale for the inactive phase is described by Darcy law for the permeation of viscous fluid in a porous medium as discussed previously^8^,^9^, whereas the characteristic time for the active phase is characterized by the condition of clogging as discussed in the present paper. Note that in the cavity regime of the present study, which is not an intermittent phase, the pressure gradient may be important for the stabilization of the suspended sand layer but it does not govern the condition of the collapse of the cavity.

The spontaneous bubbling and cavity collapse we discussed in this study may be useful for mixing a fluid and powder in industrial applications. This is expected because forced fluidization processes are essential for applications, such as petroleum refining and biomass gasification, in which bubbles created in fluidized bed reactors affect the efficiency^26^,^27^. A merit of the bubbling and cavity collapse discussed in the present study is that these phenomena occur spontaneously due to gravity and there is no need to pump a fluid inside a granular medium: efficient or violent mixing (by virtue of the bubbling or cavity collapse, respectively) is repeatedly achieved just by rotating a container. In other words, the fluidized bed reactor is driven by an externally imposed air flow, whereas the present bubbling and cavity regimes are driven spontaneously by gravity.

To conclude, the present results reveal novel aspects of granular dynamics when a suspended granular layer becomes stabilized to a certain extent and the interplay of the stabilized granular bed and clogging of the granular flow is important, which could open new avenues of research within granular physics. We have shown (1) that bubbling and cavity emerge as a result of the interplay of the clogging and the stability, (2) that the rising speed of a bubble (comparable to the growing speed of a cavity), the initial bubble size, and the constant formation of bubbles are understood by introducing a life time for a sand particle exposed to the interface of a suspended granular layer and a clogging condition resulting from the competition between two characteristic flow rates, and (3) that the sudden collapse of the suspended layer is explained by considering a force balance between gravity and solid-like friction with walls. The present results would be relevant to applications for mixing a fluid with grains.

## Methods

### Experimental

The experiment is performed with a cell composed of acrylic plates of thickness 3 mm. The height 2*H* is fixed to 162 mm, whereas the thickness *L* and the slope *α* (that defines the angle *θ*) range from 3 to 56 mm, and from 60 to 85 deg., respectively. The outlet width 2*w* of the hopper is varied from 1.5 to 3 mm. The average diameter *d* of beads is changed from 30 to 100 *μ*m (GLB-30, GLB-40, GLB-60, GLB-100, Assoc. Powder Process Ind. and Eng., Japan). We observe the transport of beads through the outlet by taking movies using a video camera (Canon, iVHS HF S21) either from the front or lateral side of the cell.

### Theory

The force *F*_fric_ can be estimated by noting that the pressure inside the granular medium is in the hydrostatic regime (the sand layer is not thick enough for Janssen’s model to be valid^28^,^29^): the pressure inside the granular medium at position *z* measured from the outlet is simply given by 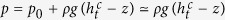. The friction force is acting from the two vertical walls and the other two walls with slope *α*. We consider a simple case *L* ≫ *H*, in which the latter contribution becomes dominant. For a wall element *Ldl* (*dl* sin *θ* = *dz*) at the the height *z*, the normal force is given by 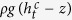: this element is subject to the friction force 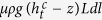 with *μ* the coefficient of the maximal static friction along the slope *α* = tan *θ* and thus the vertical component is given by 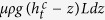. For the layer located between the position 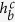 to 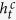 the total friction *F*_fric_ applied by the two walls of slope *α* is given by 
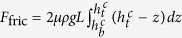
, which gives the expression used in deriving Eq. (4).

## Additional Information

**How to cite this article**: Yagisawa, Y. *et al.* Stationary bubble formation and cavity collapse in wedge-shaped hoppers. *Sci. Rep.* **6**, 25065; doi: 10.1038/srep25065 (2016).
